# Unstable chromosome rearrangements in *Staphylococcus aureus* cause phenotype switching associated with persistent infections

**DOI:** 10.1073/pnas.1904861116

**Published:** 2019-09-16

**Authors:** Romain Guérillot, Xenia Kostoulias, Liam Donovan, Lucy Li, Glen P. Carter, Abderrahman Hachani, Koen Vandelannoote, Stefano Giulieri, Ian R. Monk, Mayu Kunimoto, Lora Starrs, Gaétan Burgio, Torsten Seemann, Anton Y. Peleg, Timothy P. Stinear, Benjamin P. Howden

**Affiliations:** ^a^Department of Microbiology and Immunology, The University of Melbourne, The Peter Doherty Institute for Infection and Immunity, Melbourne, 3000, Australia;; ^b^Infection and Immunity Theme, Monash Biomedicine Discovery Institute, Department of Microbiology, Monash University, Clayton, 3800, Australia;; ^c^Doherty Applied Microbial Genomics, The University of Melbourne, The Peter Doherty Institute for Infection and Immunity, Melbourne, 3000, Australia;; ^d^Department of Immunology and Infectious Diseases, John Curtin School of Medical Research, Australian National University, Canberra, 2601, Australia;; ^e^Microbiological Diagnostic Unit Public Health Laboratory, The University of Melbourne, The Peter Doherty Institute for Infection and Immunity, Melbourne, 3000, Australia;; ^f^Department of Infectious Diseases, The Alfred Hospital and Central Clinical School, Monash University, Melbourne, 3004, Australia;; ^g^Infectious Diseases Department, Austin Health, Heidelberg, 3084, Australia

**Keywords:** *Staphylococcus aureus*, small-colony variant, chromosomal rearrangement, genome instability, restriction modification system

## Abstract

*Staphylococcus aureus* small-colony variants (SCVs) are associated with unusually chronic and persistent infections despite active antibiotic treatment. The molecular basis for this clinically important phenomenon is poorly understood, hampered by the instability of the SCV phenotype. Here we investigated the genetic basis for an unstable *S. aureus* SCV that arose spontaneously while studying rifampicin resistance. This SCV showed no nucleotide differences across its genome compared with a normal-colony variant (NCV) revertant, yet the SCV presented the hallmarks of *S. aureus* linked to persistent infection: down-regulation of virulence genes and reduced hemolysis and neutrophil chemotaxis, while exhibiting increased survival in blood and ability to invade host cells. Further genome analysis revealed chromosome structural variation uniquely associated with the SCV. These variations included an asymmetric inversion across half of the *S. aureus* chromosome via recombination between type I restriction modification system (T1RMS) genes, and the activation of a conserved prophage harboring the immune evasion cluster (IEC). Phenotypic reversion to the wild-type–like NCV state correlated with reversal of the chromosomal inversion (CI) and with prophage stabilization. Further analysis of 29 complete *S. aureus* genomes showed strong signatures of recombination between *hsdMS* genes, suggesting that analogous CI has repeatedly occurred during *S. aureus* evolution. Using qPCR and long-read amplicon deep sequencing, we detected subpopulations with T1RMS rearrangements causing CIs and prophage activation across major *S. aureus* lineages. Here, we have discovered a previously unrecognized and widespread mechanism of reversible genomic instability in *S. aureus* associated with SCV generation and persistent infections.

## Significance

*Staphylococcus aureus* is a major human pathogen known to exhibit subpopulations of small-colony variants (SCVs) that cause persistent or recurrent infections. The underlying mechanisms promoting the SCV phenotypic switching and adaptation to persistent infection are poorly understood. Moreover, the instability of this frequently reverting phenotype hampers diagnosis and study. Here we show that SCVs with reduced virulence but increased immune evasion and persistence properties can arise from reversible chromosomal instability. This mechanism of SCV generation implies an asymmetric chromosome inversion and the activation of prophage-encoding genes used for immune evasion. Assessment of major *S. aureus* lineages indicates this genomic plasticity is a common but previously unrecognized mechanism used by *S. aureus* to cause persistent and relapsing infections.

*Staphylococcus aureus* is an important human pathogen that causes a variety of community- and hospital-acquired infections, which range from harmless skin infections to severe systemic infections, such as sepsis (1). An issue with some *S. aureus* infections is their chronic and recurrent nature despite appropriate antibiotic treatment. An atypical bacterial state that manifests on solid media as small-colony variants (SCVs) is thought to be a major cause of persistent *S. aureus* infections (2). Isolation of *S. aureus* SCVs from infected patients is frequently described with an incidence between <1 and 30% (3). The SCV phenotype is often unstable and characterized by slow growth, attenuated virulence, and an increased ability to persist in host cells and evade the immune system (3). The high rate of reversion of SCVs to normal phenotypes makes both clinical identification and laboratory characterization difficult. Several stable SCV prototypes recreated in vitro by mutagenesis of genes encoding metabolic pathways or after selection by antibiotics have been thoroughly studied as a proxy of clinical SCVs (4, 5). These stable SCV mutants are commonly associated with mutations in genes involved in the electron transport chain or metabolic genes causing auxotrophy to hemin, menadione, or thymidine (6–9). It is now understood that the physiological changes between normal and SCV *S. aureus* phenotypes are more complex than reflected by defined mutants and that the generation of subpopulations of SCVs is part of the normal life cycle of *S. aureus* (10, 11). Gao et al. and Cui et al. (12, 13) described SCVs associated with a tandem chromosome duplication and inversion, respectively, the latter rearrangement an almost symmetrical flip of 1.26 Mb impacting 2 inverted copies of the pathogenicity island *SaPlm*. These chromosomal changes represent rare recombination events, and there is no evidence of conserved chromosomal changes causing reversible SCV phenotypic switching more widely. Here, we present an in-depth genomic and phenotypic characterization of an unstable SCV that reverts to a normal-colony phenotype, in the absence of a specific genome mutation or insertion/deletion. We provide evidence that *S. aureus* exploits the recombinogenic properties of the type I restriction modification system (T1RMS) to generate large and unbalanced chromosomal inversions (CIs). The *hsdMS*-mediated CIs and associated Φ*Sa3* prophage activation are important but previously unrecognized mechanisms of genetic variation in clinical *S. aureus* lineages and represent a potent mechanism for *S. aureus* populations to reversibly switch on and off persistent phenotypes.

## Results

### Isolation of SCV Subpopulations following Introduction of the Most Common Rifampicin Resistance Mutation in *S. aureus*.

While introducing the RpoB-H481N rifampicin resistance mutation in *S. aureus* (14, 15), we observed the emergence of a subpopulation of SCVs among a wild type (WT)-like normal-colony variant (NCV) morphotype (*SI Appendix*, Fig. S1). The SCV reverted to the NCV morphotype upon subcultivation in brain heart infusion (BHI) broth and produced a mixed population of SCVs and NCVs with an SCV proportion ranging from 20 to 100%. The doubling time in BHI broth was 60.8 min (±12.6 SD) versus 33.6 min for the NCV (±0.2 SD) and WT (±0.2 SD).

### Phage Activation, and Not Point Mutations, Differentiates the SCV from the NCV.

In order to investigate the genetic basis for the emergence of this unstable SCV phenotype, we sequenced the genomes of the *rpoB*-H481N-SCV, *rpoB*-H481N-NCV, and the initial WT bacteria using standard Illumina chemistry (16). Mapping of the 300-bp paired-end reads to the fully assembled reference genome of the NRS384 WT strain (17) confirmed that both the SCV and NCV carried the *rpoB*-H481N mutation, but surprisingly no additional single-nucleotide polymorphisms (SNPs) or insertion/deletion (indels) mutations differentiated the 2 colony morphotypes.

**Fig. 1.**
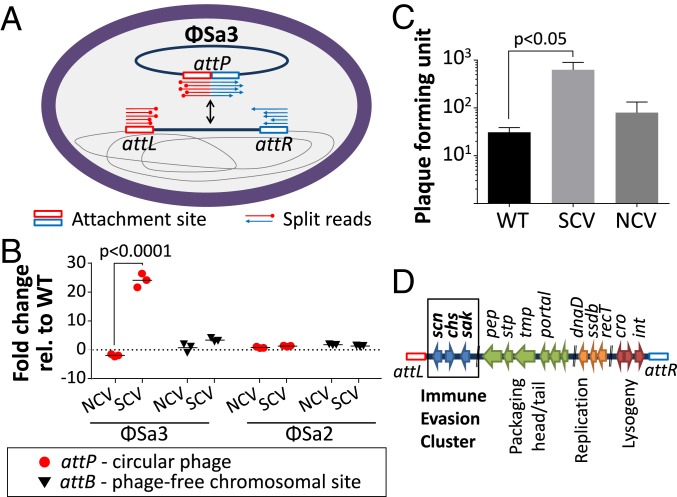
Detection of Φ*Sa3* prophage activation in the SCV. (*A*) Whole-genome sequencing using short-read technology and split-read analysis. Split reads (red and blue arrows) corresponding to a circular form of Φ*Sa3* (attP site) were uniquely detected in the SCV. (*B*) Quantification by qPCR of circular and excised forms of Φ*Sa3* and Φ*Sa2*. The relative fold change in the number of circular Φ*Sa3* phages was significantly different in the SCV compared with the WT. (*C*) Quantification of phage particles by plaque assay. The *y* axis indicates change in plaque-forming units compared with wild type, enumerated in 200 µL of filtered supernatant from overnight LB cultures. Error bars represent SD. Statistical analysis was performed comparing SCV strains with the parental strains NRS384 from triplicate experiments and using the 2-tailed Mann–Whitney *U* test, with *P* < 0.05 set for statistical significance. (*D*) Schematic representation of the genetic organization of the Φ*Sa3* prophage. Genes are represented by arrows that are colored according to functional modules. An atypical characteristic of the Φ*Sa3* prophage is to encode 3 genes implicated in immune modulation that form the immune evasion cluster: *scn* encoding the staphylococcal complement inhibitor, *chp* encoding the chemotaxis inhibitory protein of *S. aureus*, and *sak* encoding the staphylokinase.

We then investigated chromosomal structural variations as a potential cause of the phenotype. We assessed the genome sequence data for aberrant read coverage, discordant read pairs, and split reads and found reads corresponding to the *attP* site of a circular form of the prophage Φ*Sa3*. These reads were not detectable in either the NCV or WT as a distinctive feature of the SCV (Fig. 1*A*). Quantitative PCR analysis showed activation of the Φ*Sa3* prophage in the SCV, with a 25-fold increase in the circular form of the phage (*P* < 0.0001; Fig. 1*B*). Phage circularization in the SCV was not associated with an increase of the phage-free chromosomal site (*attB*) of the phage (Fig. 1*B*), indicative of phage replication. We also tested the activation of the Φ*Sa2* prophage and did not observe any change in *attP* or *attB* copy number.

To further confirm Φ*Sa3* prophage activation, we measured the number of phage particles by plaque assay. The results confirmed a significant increase in phage particle production in the SCV (*P* < 0.05; Fig. 1*C*). Interestingly, the prophage Φ*Sa3* harbors the immune evasion cluster (IEC) genes that can favor the persistence of the SCV during infection, with 3 genes promoting immune evasion: *chp* (chemotaxis inhibitory protein of *S. aureus*; CHIPS), *scn* (staphylococcal complement inhibitor; SCIN), and *sak* (staphylokinase) (Fig. 1*D*) (18–21), suggesting that phage activation in the SCV may be promoting persistent infection through enhanced expression of these factors.

### The SCV Subpopulations Share Phenotypes Commonly Associated with Isolates from Persistent Infections.

**Fig. 2.**
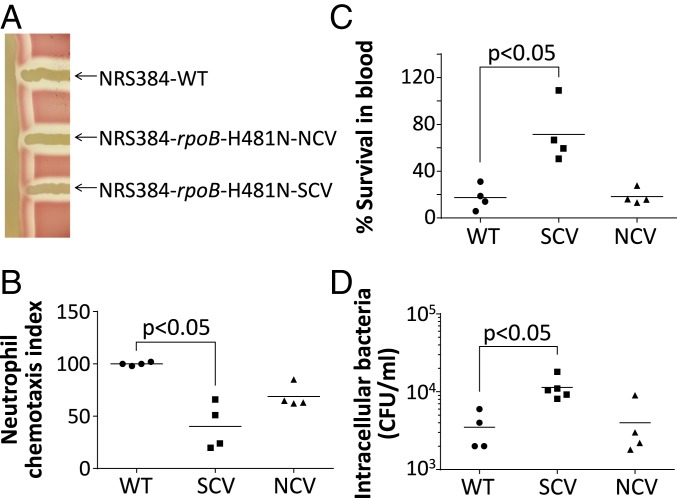
Phenotypic characterization of the *S. aureus* SCV. (*A*) Hemolysin activity of *S. aureus* NRS384 WT, NRS384-*rpoB*-H481N-NCV, and NRS384-*rpoB*-H481N-SCV. Reduction in delta-hemolysin activity in the SCV is visible by reduced hemolysis at the intersection with the vertical streak of NRS384-ΔΦ*Sa3*. Reduction in alpha-hemolysis of the SCV is visible at the top and bottom of the SCV streak. (*B*) Whole human blood killing assay. The SCV is significantly less susceptible to killing by whole human blood. (*C*) Human neutrophil chemotaxis assay. The SCV shows significantly reduced neutrophil chemotaxis relative to the WT. (*D*) Host cell invasion assay. Significantly more intracellular SCV was recovered 2 h after infection of A549 adenocarcinoma human alveolar basal epithelial cells. All experiments were performed in at least quadruplicate and horizontal bars represent the mean. A 2-tailed Mann–Whitney *U* test with *P* < 0.05 was used to infer statistical significance. CFU, colony-forming unit.

Consistent with clinical SCV isolates, we found a reduction in alpha-hemolysis and delta-hemolysis in our rifampicin-resistant SCV (Fig. 2*A*), suggesting a down-regulation of key virulence factors. In human-adapted *S. aureus* strains, the beta-hemolysin gene is interrupted by the immune evasion cluster encoding prophage Φ*Sa3* (22). We did not observe any beta-hemolytic activity, confirming our qPCR results, showing that the significant increase of circular phage copies in the SCV is not associated with Φ*Sa3* chromosomal excision and recapitulation of the beta-hemolysin gene.

We then assessed the immune evasion phenotype of the SCV in a whole-blood killing assay. The SCV was significantly more resistant to whole-blood killing, with ∼70% SCV survival after a 4-h exposure compared with ∼20% survival for the WT and the NCV (*P* < 0.05; Fig. 2*B*). Among the innate immune cells, neutrophils are considered critical for clearing *S. aureus* infections. Interestingly, it is the IEC-encoding phage that is solely activated in the SCV, and the IEC harbors the gene encoding the CHIPS, a potent inhibitor of neutrophil chemotaxis (18). Therefore, we assessed neutrophil chemotaxis ex vivo with purified human neutrophils and found that the SCV attracted neutrophils significantly less than the WT strain (*P* < 0.05; Fig. 2*C*).

Another important characteristic commonly associated with SCVs and persistent *S. aureus* is their capacity to invade and persist in host cells. We found that after 2 h of infection, A549 cells contained significantly more SCV than WT or NCV bacterial cells, with a significantly increased propensity of the SCV to invade cells (*P* < 0.05; Fig. 2*D*). We did not observe any difference in bacterial load at 24, 48, or 96 h, suggesting that the difference in cell invasion does not correlate with a difference in long-term intracellular persistence in vitro.

### The SCV Subpopulation Demonstrates Major Transcriptional Changes Compared with NCV and WT *S. aureus*.

To gain a deeper understanding of the changes that were impacting SCV interactions with the host, we used RNA-seq to assess changes in gene expression at a genome-wide level in the SCV and NCV versus WT *S. aureus* (16). The results showed that the SCV subpopulation was affected by major transcriptional changes whereas, in striking comparison, the NCV did not show any significant change in gene expression when compared with the WT strain (*SI Appendix*, Fig. S2*A*). This result aligns with our previous observation that the *rpoB*-H481N-NCV has no fitness cost in rich media when compared with the WT parental strain (14). In the SCV a total of 207 genes were differentially expressed, with 86 genes up-regulated and 121 genes down-regulated (false discovery rate; log2 expression fold change > 1; *SI Appendix*, Fig. S2*A* and Dataset S1). Although the IEC genes encoded by Φ*Sa3* did not appear up-regulated in the condition tested, 30 Φ*Sa3* genes were among the most up-regulated genes, together with ribosomal protein-encoding genes and genes associated with glycolysis. We also found virulence down-regulation in the SCV, with a significant decrease in expression of the major *S. aureus* virulence regulon *agr*. The down-regulation of the *agr* quorum-sensing system and the effector molecule RNAIII is known to be associated with persistent infection and SCVs (11). We found that agr/RNAIII regulatory system together with its regulated genes encoding phenol-soluble modulins (beta-1 and beta-2) and hemolysins alpha and delta represent the most down-regulated genes in the SCV (*SI Appendix*, Fig. S2*B* and Dataset S1). While agr/RNAIII is likely playing an important role in the SCV global transcriptional changes of the SCV, we identified 19 other regulators that are significantly differentially expressed in the SCV (*SI Appendix* and Dataset S1). Among these genes, the decreased expression of the *cspA* regulator that positively regulates the biosynthesis virulence factor staphyloxanthin responsible for the *S. aureus* yellow pigmentation (23) could explain the characteristic depigmentation of the SCV. We also observed a reduced expression of ribosomal protein genes, *spa* (encoding protein A), type VII secretion system, amino acid metabolism, and other transporters. Finally, several genes encoding adhesins were down-regulated in the SCV (*efb*, *clfB sasA*, *sasG*, *sdrC*, *sdrE*) with the exception of the fibronectin-binding protein A gene (*fnbA*), which was up-regulated. FnbpA has been shown to be essential for internalization into host cells, a feature consistent with our increased A549 invasion results and the intracellular lifestyle of SCVs (24–26).

All of the transcriptional changes observed in the *rpoB*-H481N-SCV were consistent with commonly observed characteristics of clinical SCVs (increased glycolysis, down-regulation of virulence factors, including reduced hemolysin secretion, reduced pigmentation, and enhanced propensity to be internalized in nonphagocytic cells), as well as the phenotypic changes we observed in this strain, described above (25–27).

### Long-Read DNA Sequencing Reveals an Asymmetric Inversion of Half the SCV Chromosome.

**Fig. 3.**
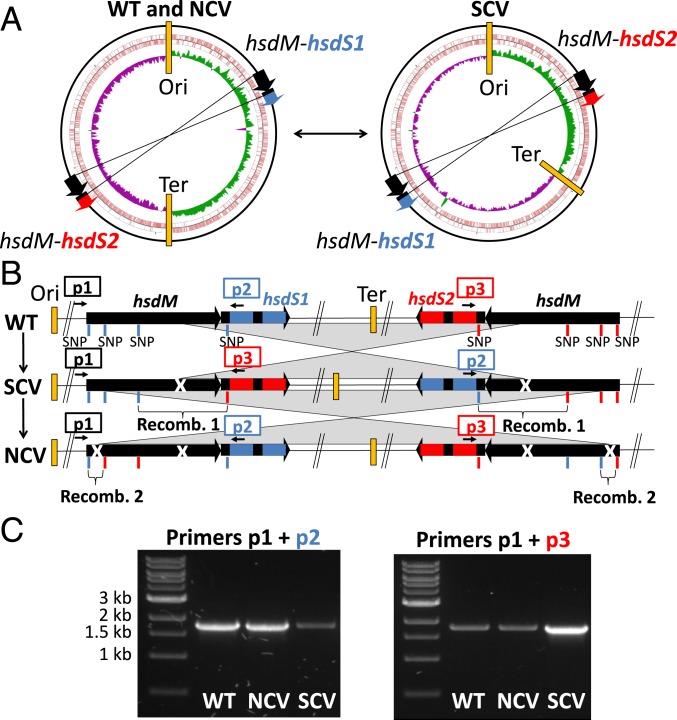
Detection of *S. aureus* asymmetric chromosomal inversion by long-read whole-genome sequencing. (*A*) Chromosome map of complete genomes. The conformations of the WT and *rpoB*-H481N-NCV (*Left*) and SCV (*Right*) circular chromosomes are represented. The SCVs have a 1.41-Mb CI. The inner circles represent GC skew of the leading strand (green) and lagging strand (purple). The CI in the SCV is asymmetric as it displaces the replication terminus (Ter) and generates a longer lagging-strand replichore (left arm) compared with the leading-strand replichore (right arm). The circles with red represent coding sequence on the forward strand (outer circle) and reverse strand (inner circle). (*B*) Sites of chromosomal recombination breakpoints in the SCV and NCV. The homologous inverted sequences of the *hsdMS* loci are indicated in black. Representative SNPs of the *Right* and *Left hsdMS* loci are indicated by blue and red vertical lines below the genic maps. Recombination 1 region leading to the SCV inverted chromosome and recombination 2 leading to the reversion of the SCV-to-NCV noninverted chromosome were identified by the modification of the SNP patterns. (*C*) Confirmation of CI by PCR. The PCR primer pairs p1 + p2 detect the noninverted chromosomal conformation (WT and NCV) and primer pairs p1 + p3 detect the inverted chromosomal conformation detected in the SCV as indicated in *B*.

Given the substantial phenotypic and transcriptomic changes observed in the SCV in the absence of explanatory point mutations, we next compared structural variation of the SCV and NCV chromosomes using complete genome sequences (16). We confirmed that NCV chromosome structure was identical to the previously fully assembled WT strain. Notably, however, the SCV chromosome contained a large 1.42-Mb inversion, caused by recombination between homologous and inverted copies of the methylase (*hsdM*) and specificity subunit (*hsdS*) genes of a T1RMS (Fig. 3*A*).

The SCV CI arose inside nearly perfect inverted repeats of DNA sequences of 1,748 bp spanning the T1RMS. This inversion was not detectable using standard Illumina sequencing because the length of the homologous loci exceeded both fragment length and read length. Complete genome comparisons also revealed 22 differentiating SNPs dispersed along the longest conserved region of the inverted *hsdMS* locus. Due to sequence alignment ambiguity between the 2 nearly identical loci, these SNPs were not identified by mapping the Illumina reads to a reference genome. Detailed comparison of the *hsdMS* SNP pattern of the SCV, NCV, and WT allowed us to identify the recombination breakpoints. This high-resolution comparative genome analysis showed that a single recombination event is sufficient to explain the SCV inversion from the WT isolates (recombination 1; Fig. 3*B*). The mosaic SNP pattern in the *hsdMS* copies shows that the NCV isolates resulted from a reversion of the SCV CI back to a WT form.

In order to assess if the global transcriptional change could be linked to this major CI, we mapped all differentially regulated genes across the chromosome (*SI Appendix*, Fig. S2*C*). We found that genes displaced by the inversion that are either closer or distal to the origin of replication by the inversion exhibited a significant enrichment of up- and down-regulated genes, respectively (*C-ori*, *F-ori* loci; *SI Appendix*, Fig. S2*C*). This observation supports the idea that a replication-associated gene-dosage effect is impacting the transcriptional changes to create the SCV, as gene copy number increases closer to the origin during replication (28, 29).

To further confirm that the SCV phenotype was associated with the inversion of the chromosome, we designed primers spanning the homologous inverted repeats and specific to either the WT/NCV or SCV chromosomal conformations (Fig. 3*B*). SCVs were consistently associated with readily detected amplicons diagnostic for the CI and NCV, and the WT had amplicons corresponding to the WT chromosomal conformation. We also found that reversion of the SCV to NCV was always associated with the restoration of the inversion to the WT chromosome form. Unexpectedly, a faint PCR amplicon corresponding to the SCV inversion was repeatedly obtained in the WT and NCV, indicating the presence of rarer subpopulations of SCV-like CIs in both the WT and NCV *S. aureus* cultures used for genomic DNA extraction. Similarly, the chromosome restored to its WT/NCV form was identifiable by a faint amplicon in the SCV, suggesting that the SCV–NCV switching represents a phase-variable mechanism (Fig. 3*C*).

### Chromosomal Inversion and Phage Activation Represent a Conserved Mechanism Promoting Reversible SCV Phenotypic Switching in Clinical *S. aureus*.

To investigate whether the CI mediated by the *hsdMS* alleles is a conserved mechanism of reversible SCV generation, we screened 29 fully assembled genomes of the most prevalent *S. aureus* lineages for conserved, inverted repeats larger than 1 kb. This analysis identified 27 loci potentially promoting CIs (*SI Appendix*, Table S1). With a conservation among 26 of the 29 fully assembled genomes available, the 2 inverted copies of the *hsdMS* loci of the T1RMS represented the most conserved inversion hotspots after ribosomal RNA loci in *S. aureus* (*SI Appendix*, Table S1). Inverted copies of the *hsdMS* loci were detected among 11 different sequence types (ST8, ST239, ST5, ST1, ST105, ST133, ST250, ST151, ST93, ST36, and ST254) and absent only in 2 (ST22 and ST398). The next most prevalent loci potentially promoting CI were the mobile elements *IS1182* and *IS1181* found in 7 and 6 different genomes, respectively. We also identified 2 previously described inversion loci described in *S. aureus* strains Mu50 and OC8 (13, 30). These 2 loci correspond to the mobile elements *IS256* and *SaPlm* in inverse orientation and represent potential inversion loci in only 3 and 2 strains, respectively.

Similarly, we investigated the conservation of the Φ*Sa3* prophage in a global database of 7,099 *S. aureus* genomes (14). We found Φ*Sa3* in more than 80% of *S. aureus* strains (5,711/7,099), and the proportion of strains encoding the Φ*Sa3* prophage among the most prevalent STs shows a high level of conservation among human-associated *S. aureus* lineages (*SI Appendix*, Table S2).

**Fig. 4.**
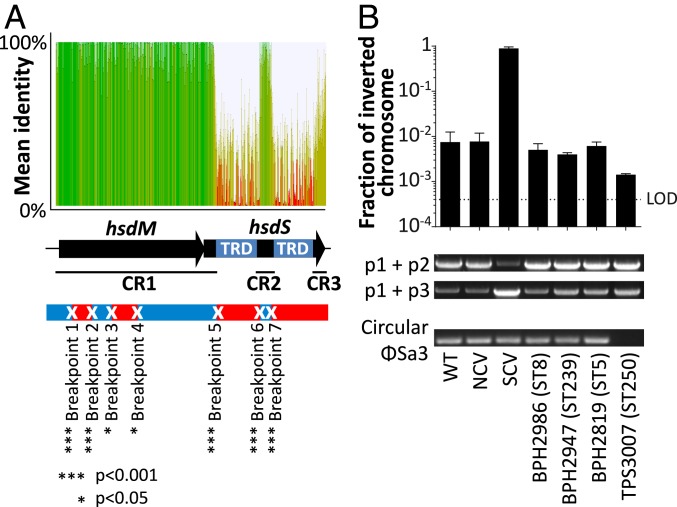
Detection of chromosomal recombination and inversion along the inverted copies of the *hsdMS* loci in different *S. aureus* lineages. (*A*) Detection of recombination breakpoint signals along *hsdMS* inverted copies of 29 complete *S. aureus* genomes. The *hsdMS* locus is composed of 3 conserved regions (CR1 to CR3) and 2 nonconserved regions that correspond to the target recognition domain (TRD) responsible for methylation site specificity. Five significant signatures of recombination in CR1 and 2 signatures in CR2 were identified by the Genetic Algorithm for Recombination Detection (31). (*B*) Detection of the circular phage and quantification of the CI by qPCR in different *S. aureus* backgrounds. The bar plot represents the fraction of inverted chromosomes calculated from the absolute quantification by qPCR of p1 + p2 (WT chromosomal conformation) and p1 + p3 (SCV-associated CI). Columns and error bars represent mean and SD of 3 replicates. The limit of detection (LOD) is indicated by a dotted line. PCR amplicons corresponding to noninverted chromosome (*Upper* gel), inverted chromosome (*Middle* gel), and circular form of the prophage Φ*Sa3* (*Lower* gel) are indicated below the bar plot.

We speculated that if the inverted copies of *hsdMS* loci represented a conserved recombination hotspot promoting the generation of SCV subpopulations in *S. aureus*, molecular signatures of frequent recombination should be detectable along the sequence. To do so, we exploited nucleotide conservation along all inverted copies of the *hsdMS* loci to scan for signatures of past recombination. The Genetic Algorithm for Recombination Detection identified 5 significant recombination breakpoints in conserved region 1 (CR1) of the locus and 2 in conserved region 2 (CR2) (Fig. 4*A*) (31), showing that these inverted homologous genes have recombined several times throughout *S. aureus* evolution.

We then assessed if CIs were detectable by absolute quantitative PCR using genomic DNA extraction of overnight cultures of 4 different *S. aureus* clinical isolates of different lineages. We found a significant fraction of CIs in 4 different *S. aureus* strains representing the globally common sequence types 5, 8, 239, and 250 (Fig. 4*B*). We also detected circular forms of the Φ*Sa3* prophage in ST5, ST8, and ST239 clonal backgrounds (Fig. 4*B*). The only strain where the Φ*Sa3* circular form was not detected in *S. aureus* was TPS3007 (ST250), which lacks Φ*Sa3*.

**Fig. 5.**
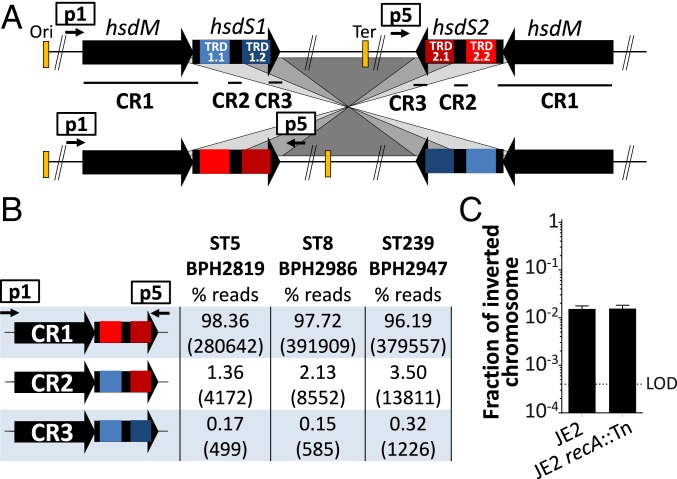
(*A*) Paralogous copies of the T1RMS are substrates for the chromosomal inversion. (*B*) The entire right-hand *hsdMS* locus corresponding to the inverted chromosome was amplified by PCR from ST5, ST8, and ST239 *S. aureus* lineages using primers p1 + p5 and deep-sequenced with Nanopore long-read technology. Three different inverted-repeat recombination events were detected corresponding to CR1 or CR2 or CR3 in 3 different *S. aureus* lineages at different frequencies as estimated by read abundance. Numbers in the table in parentheses indicate the total number of reads mapping to each of the 3 recombination structures. (*C*) Absolute quantification by qPCR of inverted chromosomes in the *S. aureus* JE2 strain and JE2 transposon mutant of the *recA* gene from the Nebraska transposon library. Columns and error bars represent mean and SD of 3 replicates.

Interestingly, T1RMSs have been shown to promote short-range CIs at a high rate in many different bacterial species such as *Mycoplasma pulmonis*, *Listeria monocytogenes*, *Streptococcus suis*, and *Bacteroides fragilis* (32). In these species, the 3 conserved regions of inverted alleles of *hsdS* genes recombine to rearrange the target recognition domain and therefore modify the methylation specificity of the T1RMS and epigenetic regulation. In order to investigate if the same type of inversion was occurring in *S. aureus*, we assessed by long-read amplicon deep sequencing the inverted form of the *hsdMS* loci of 3 different strains corresponding to 3 different *S. aureus* lineages (ST8, ST5, ST239) (16). We found that not only the CR1 region of the *hsdMS* loci generates an inversion (CR1) but also the internal conserved region of *hsdS* (CR2) and its 3′ end (CR3) in the 3 different lineages tested (Fig. 5*A*). These 3 rearrangements occurred at different frequencies that were conserved between the 3 sequence types we examined, around 98, 2, and 0.2% for each of CR1, CR2, and CR3, respectively (Fig. 5*B*). Furthermore, we found that similar to the small CI promoted by other T1RMSs, the CI identified in the SCV is not impacted by *recA* inactivation, and as a result is not mediated by homologous recombination (Fig. 5*C*) (33, 34). These data suggest that depending on the site of inversion, hybrid methylomes might be generated, if recombination within CR2 occurs.

## Discussion

Here, we have uncovered the role of reversible chromosomal rearrangements in promoting an unstable SCV that has the phenotypes typical of clinical SCVs. We have shown that this mechanism occurs in globally important *S. aureus* clones, thus providing a potential explanation for the elusive and transient nature of this clinical phenomenon. We found that the SCVs exhibit reduced neutrophil chemotaxis and higher resistance to human blood killing (Fig. 2). The transcription profile of the SCV was also similar to those described for SCVs isolated from chronically infected patients (10, 11, 27). Several gene expression changes could be linked to features of persistent infection: reduced alpha- and delta-hemolysin gene expression, increased expression of *fnbA* (cell adhesion), and reduced expression of staphyloxanthin synthesis genes (reduced pigmentation).

Our comparative chromosome analysis of SCVs with NCV revertants revealed that these global transcriptional changes correlate with 2 major genomic rearrangements undetectable by classical short-read sequencing, namely the activation of prophage-encoding immune evasion genes and the inversion of half of the chromosome through the recombination of inverted copies of the *hsdMS* loci of a T1RMS (Figs. 4 and 5). We found that both phage activation and CI can quickly revert to a “wild-type” conformation, which resumes the wild-type NCV status both transcriptionally and phenotypically. The discovery that SCVs with persistent phenotypes are generated by phase-variable chromosome structural variants at conserved recombination hotspots of instability represents an important mechanistic understanding of SCV generation and *S. aureus* pathogenesis.

CIs have been shown to impact gene expression in several ways: 1) by modifying gene orientation relative to replication; 2) by affecting gene position and therefore gene dosage (increased gene copy number closer to the replication origin during replication); and 3) by disruption of genetic organization at the 2 sites of recombination (28, 29). The inversion we describe here does not modify gene orientation relative to replication, but it does change gene dosage and so significantly impacts the transcriptome (*SI Appendix*, Fig. S2). As several transcriptional regulators are displaced relative to the replication origin, the impact on global gene expression will extend beyond those genes within the inverted region. In *Bacillus subtilis*, mutants with asymmetrical CIs showed significantly slower growth than the wild-type strain. Decreased growth rates caused by disruption of the *Ori*–*Ter* axis were also reported in other bacteria and can be attributed to the net imbalance of the different lengths of the 2 replichores (35). Therefore, asymmetrical CIs are an efficient mechanism to slow bacterial growth and generate SCVs.

Genome alterations and phage mobilization during *S. aureus* infection have been reported previously (36–38), and the chromosomal rearrangements described here are not the first examples of genomic rearrangements associated with SCV generation (12, 13). Cui and collaborators (13) identified a symmetrical inversion of 1.26 Mb impacting 2 inverted copies of the pathogenicity island *SaPlm*. Our exhaustive screen for potential hotspots of inversion revealed that these loci are not universally conserved in *S. aureus* (*SI Appendix*, Table S1). This specific inversion is therefore not responsible for SCV generation in the majority of *S. aureus* lineages.

Despite most Φ*Sa3* genes appearing overexpressed in the SCV in our transcriptomic data, the chemotaxis inhibitory protein (CHIPS)-encoding gene and the other IEC genes were not significantly up-regulated. Nevertheless, as it has been previously shown that an increase in Φ*Sa3* copy number is accompanied by increased expression of the IEC genes (39) and that the IEC genes are coregulated by different chromosomal loci (40), therefore it is likely that when activated in the context of immune selective pressure, the high copy number of IEC genes enables an increased expression level. Interestingly, the SCV characterized by Gao et al. (12) is also characterized by the activation of the Φ*Sa3* prophage with the IEC (40). Noticeably, a shared characteristic between these SCVs associated with chromosomal rearrangement is the presence of the rifampicin resistance mutation in *rpoB* affecting residue H481, which suggests an interplay with rifampicin selective pressure and/or with the modification of the RNA polymerase.

A distinctive characteristic of the rearrangement described here is its high conservation across a range of *S. aureus* lineages. The IEC-encoding phage represents the most prevalent phage in human-adapted *S. aureus* strains (41). Likewise, the copies of the *hsdMS* loci that promoted the CIs are conserved in their inverse orientation in most *S. aureus* lineages despite apparently functional redundancy with respect to HsdM. We have shown previously that HsdM1 or HsdM2 can functionally complement the deletion of either gene (17). Previous comparative genomic studies of different bacterial species suggested that CIs are a common feature of bacterial genome evolution (42). The conservation of hotspots of chromosomal instability in *S. aureus* might have been maintained throughout evolution to confer a bet-hedging strategy against adverse conditions, such as strong immune or antibiotic selective pressures. Furthermore, the genetic mechanisms described here are reversible and can promote a phenotypic switching from an SCV-persistent form to the NCV more virulent form, therefore providing a mechanistic explanatory model for relapsing infections. In *S. aureus*, the T1RMS is thought of as a fixed barrier which defines clonal complexes; however, the identification of recombination between *hsdS* alleles (from the Nanopore sequencing; Fig. 5) suggests that it has the propensity to generate both SCV and hybrid methylomes. Changes in the methylome may impact on the uptake of DNA and epigenetic regulation of the SCV.

Surprisingly, SOS response genes such as *recA* and *lexA* were not activated in the SCV, suggesting that SOS response was transient following the inversion or that Φ*Sa3* activation is not induced by a DNA damage stress caused by the CI. The factors promoting Φ*Sa3* induction remain to be discovered. One possibility is that inversion-mediated up-regulation of the putative Φ*Sa3* antirepressor gene (NRS384_2027; *SI Appendix* and Dataset S1) (41) leads to phage activation. Alternatively, the inversion may be a consequence of Φ*Sa3* activation, as the Φ*Sa3* integrase is a close homolog of the tyrosine recombinases that promote small T1RMS-mediated inversions in other bacterial species (32).

An important conclusion from our work is that structural variation among clinical SCV isolates cannot be detected by classical short-read genome sequence analysis and likely remained undetected among previously sequenced SCVs. Long-read sequencing technologies as we have deployed will need to be used to address this shortcoming. A parallel can be made here with cancer genomics, where chromosome rearrangements are a hallmark of most cancers (43, 44). Our results support a model of a commensal bacterium undergoing reversible chromosomal rearrangements that disrupt homeostasis with its host to cause persistent infections.

## Materials and Methods

*SI Appendix*, *Materials and Methods* describes in detail the materials and procedures used in this study, including bacterial culture conditions, short- and long-read genome sequencing, quantitative PCR, phenotypic assays, RNA-seq, analysis of conservation of Φ*Sa3* and inverted repeats among *S. aureus* genomes, and statistical analysis.

## Supplementary Material

Supplementary File

Supplementary File
