# Supplementary material for: Unstable chromosome rearrangements in Staphylococcus aureus cause phenotype switching associated with persistent infections
Source: Proc Natl Acad Sci U S A. 2019 Sep 16;116(40):20135–40. doi: 10.1073/pnas.1904861116 (PMC6778178; doi:10.1073/pnas.1904861116)
Supplement: Supplementary File [file pnas.1904861116.sapp.pdf]

Supplementary Information for

Unstable chromosome rearrangements in *Staphylococcus aureus* cause phenotype switching associated with persistent infections

Romain Guérillot, Xenia Kostoulias, Liam Donovan, Lucy Li, Glen P Carter, Abderrahman Hachani, Koen Vandelannoote, Stefano Giulieri, Ian R Monk, Mayu Kumimoto, Lora Starrs , Gaétan Burgio, Torsten Seemann, Anton Y. Peleg, Timothy P. Stinear, Benjamin P. Howden

Benjamin P. Howden

Email: bhowden@unimelb.edu.au

This PDF file includes:

Materials and Methods

Fig. S1 to S2

Tables S1 to S4

Supplementary References

Other supplementary materials for this manuscript include the following:

Dataset S1

www.pnas.org/cgi/doi/10.1073/pnas.1904861116

MATERIALS AND METHODS

Bacterial culture condition

S. aureus strains were routinely cultivated at 37 °C in Brain Heart Infusion (BHI) broth with shaking at 200 rpm. To ensure the SCV maintained the small colony phenotype, an aliquot of the culture was diluted and plated on BHI agar plates prior to experimentation. At least 100 colonies were visually inspected. SCV cultures that reverted colonies to the WT morphotype were discarded.

Genome sequencing

Genomic DNA was isolated from overnight culture in BHI broth with DNeasy Blood and Tissue Kit (Qiagen). Before DNA extraction, harvested cells were treated with lysostaphin (Ambi) added to the Gram-positive lysis buffer (final concentration 100 µg/mL) and incubated at 37 °C for 30 min. Short read sequencing was performed on an Illumina MiSeq platform using Nextera XT libraries (300bp paired ends) according to the manufacturer instruction. Long read sequencing was performed on a PacBio RSII instrument as described previously (1).

Quantitative PCR

Quantitative PCR were performed from genomic DNA extracted from 1mL of overnight culture in BHI. qPCR was performed with the primer pairs as described in Table S3. Phire Hot Start II DNA polymerase (Thermo Fisher) was used in the qPCR and Evagreen dye (Biotium) was added into reactions in 1:20 ratio. The PCR was cycled at 98 °C for 5s, 52 °C for 10s and 72 °C for 30s. Relative quantification of circular and excised form of *ΦSa3* were performed with the ΔΔCt method using primers amplifying the chromosomal gene *dnaA* as reference and the WT strain as comparator. Absolute quantification of the chromosomal inversion was performed by generating “inversion”

and “non-inversion” standard curves generated from 10-fold serial dilution of gel purified amplicons corresponding to the inverted or non-inverted chromosomal loci. In order to control for potential PCR hybrid formed during PCR, a limit of detection was determined by using as DNA template a 1 to 1 ratio of a non-recombined *hsdM1* and *hsdM2* purified amplicons obtained from the WT strain. All qPCR experiments were performed in triplicate.

RNA-seq transcriptomic analysis

RPMI 1640 (Gibco) media were inoculated with overnight RPMI 1640 cultures of WT, NCV and SCV to a starting adjusted to OD_{600} of 0.02. Cultures were grown to mid-exponential phase (OD600 = 0.6) before 5 mL of each culture was mixed with 5 mL of RNAprotect bacteria reagent (Qiagen) and incubated for 10 min at room temperature. Bacterial cells were pelleted by centrifugation at 3300 xg for 20 min at room temp. Total RNA were extracted from the bacterial pellets using FastRNA pro blue kit (MP Biomedicals) using manufacturer's protocol with modifications. Cells were resuspended in 1 mL of RNA pro solution and lysed using Bertin Precellys 24 homogenizer set at 6000 rpm for 40 sec followed by phenol/ chloroform extraction. Samples were ethanol precipitated overnight at -20C with 0.3M sodium acetate. Samples were pelleted and dried at room temp before resuspending in DEPC treated water which were then treated with TURBO DNase (Ambion) followed by clean up with RNeasy kit (Qiagen). The absence of DNA contamination was checked by PCR and RNA integrity and purity was checked with a bioanalyzer RNA kit (Agilent). Ribosomal RNAs were depleted by Ribo-Zero rRNA Removal Kit (Illumina). Four sequencing libraries from independent RNA extraction were made for each of the WT, NCV and SCV strains using the TruSeq Stranded mRNA Library Prep Kit (Illumina) and sequenced on a single lane of an Illumina Hiseq 2500 platform (single end 100bp reads). Reads data are available on NCBI under BioProject PRJNA527676. The abundance of transcripts per genes were obtained with the kallisto

program v0.44.0 (12). Differential gene expression analysis was performed with Degust (http://degust.erc.monash.edu/) using the four RNA-seq replicates of each strains.

Conservation of *ΦSa3* and inverted repeats among *S. aureus* genomes.

A global dataset of 7099 *S. aureus* genomes previously compiled was used to estimate *ΦSa3* conservation (2). The DNA sequence of the *ΦSa3* integrase and the three IEC genes SCIN, CHIPS and staphylokinase genes were detected among the 7099 genome assemblies using abricate v0.8.11 (https://github.com/tseemann/abricate). Genomes where the integrase plus at least two genes of the IEC were detected were considered as having *ΦSa3.* The 29 fully assembled *S. aureus* genomes used to detect potential loci potentially promoting chromosomal inversion are listed in S Table S4. Inverted repeats at least 1000 bp and with more than 95% identity have been detected with a custom script using nucleotide blast (https://github.com/rguerillot/scripts/blob/master/inversion_scan.py).

Statistical analysis

Two-tailed Mann Whitney tests were performed with GraphPad Prism 7 (GraphPad Software). RNA-seq statistical analysis was performed using the limma/voom method as implemented in the Degust program (3).

Short read whole genome sequence data analysis.

SNP/indel calling were performed using the snippy pipeline v3.0 (T. Seemann, https://github.com/tseemann/snippy) and the fully assembled genome of NRS384 (4). Structural variants were investigated by manually comparing the coverage of all reads, split reads or discordant reads of the WT, NCV and SCV strains aligned onto the NRS384 reference genome.

Sequencing reads were mapped with bwa mem v0.7.17-r1188 (5) and alignment file were converted to sorted bam file using samtools v1.9. Then split reads and discordant were extracted from the bam alignment files using the extractSplitReads_BwaMem script (https://github.com/arq5x/lumpy-sv/blob/master/scripts/extractSplitReads_BwaMem) and the samtools command "samtools view -b -F 1294", respectively. The different bam alignments file of the different strains were then compared using the artemis software (6).

Long read whole genome sequence data analysis

De novo assembly were performed using HGAP2 algorithm**.** To remove remaining SNP/Indel errors, the resulting fully assembled genomes were polished with the corresponding short read sequences using snippy and then annotated with prokka v 1.13.3 (7). The final assembly of NRS384-WT, NRS384-*rpoB*-H481N-NCV and NRS384-*rpoB*-H481N-SCV and reads data are available on NCBI under BioProject PRJNA527676.

Phage plaque assay.

Phage particles of the WT, NCV and SCV strains were obtained from supernatant of overnight cultures grown at 37°C in Luria Bertani (LB) broth (Oxoid). Cultures were centrifuged at 20,000*xg* for 10 min at 4°C and the supernatant filter sterilised through 0.22 µm filters (Merck) to obtain phage extracts. Phage extracts were serial diluted in LB and 200 µl of the dilutions were mixed with 300 µL of an overnight culture of *S. aureus* RN4420 (devoid of phage) pelleted and resuspend in LB supplemented with 10mM $CaCl_2$. The infection tubes were incubated for 30 min at room temperature and then 10 mL of molten LB (3.5 g/L of agar no. 1 (Oxoid), $CaCl_2$ 10mM) were added. The molten agar was then poured over LB agar plates (15 g/L of agar no. 1 (Oxoid), $CaCl_2$ 10mM)

and the plate incubated at 37°C overnight. The numbers of Plaque Forming Unit (PFU) per 200 µL of filtered supernatants were then counted for WT, NCV and SCV stains.

Hemolytic activity

Hemolytic activity was measured on sheep blood agar plate by cross-streaking the WT, NCV and SCV strains perpendicularly to the *S. aureus* NRS384-Δ*ΦSa3* strain, which produces beta-hemolysin. The deletion of *ΦSa3* from NRS384 was constructed by allelic exchange with pIMAY-Z (4). A deletion construct was amplified by PCR with primers IM119/IM120 on genomic DNA of a spontaneous beta hemolytic clone of NRS384. The product was SLiCE cloned into pIMAY-Z (8) and transformed into *E. coli* IM08B. The purified plasmid was electroporated into NRS384 and allelic exchange conducted as detailed previously (4). As beta-hemolysin enhances lysis by delta-haemolysin but inhibits lysis by alpha-hemolysin (9), delta-hemolysin activity is visible at the intersection of the streaks after 24 hours of incubation at 37°C.

Whole human blood killing assay and human neutrophil chemotaxis assay

Whole blood was collected from healthy volunteers into EDTA tubes. Whole blood killing assays were conducted as described in (10). Neutrophils were isolated using the EasySep Direct Human Neutrophil Isolation Kit (Stem Cell Technologies), according to manufacturer's instructions. Isolated neutrophils were resuspended in Hanks' Balanced Salt solution (HBSS) and 2.5 x 10^5 neutrophils were seeded into the upper chamber of 96 well transwell plates (3µm pore size, Corning Inc). The lower chambers were seeded with either *S. aureus* at an MOI of 100, 100 nM N-Formylmethionine-leucyl-phenylalanine (FMLP) as a positive chemotaxis control or HBSS as a negative control. Plates were incubated at 37°C in 5% CO_2 for 45 min. Neutrophil chemotaxis was

measured as previously described (11). This work was conducted with approval from the Monash University Human Research Ethics Committee (approval no. CF15/3596-2015001553).

Host cell invasion assay

Overnight bacterial cultures in BHI broth were stored in 30% glycerol at -80°C prior to infection. Three stocks of each strain were assayed for CFU/ml, and the average CFU/ml for each strain was used to calculate MOI volumes. The day of infection glycerol stocks were thawed at room temperature and bacterial cells were washed once in PBS and twice in Ham's F-12K (Kaighn's) serum-free media (Thermo Fisher). One day prior to infection, the adenocarcinomic human alveolar basal epithelial cells A549 were seeded at a density of $1x10^5$ cells per well in a 24-well plate. Cells were maintained in Ham's F-12K supplemented with 10% heat-inactivated bovine serum with penicillin and streptomycin (final concentration 500 μg/ml; complete media) and incubated at 37°C in 5% CO_2 overnight. On the day of infection, one well was sacrificed for counting, while the remaining wells were washed twice with PBS, before the addition of 500 μl serum-free Ham's F-12K (Kaighn's). Cell count on the day and average CFU/ml were used to calculate volumes for an MOI of 1. Sample wells were inoculated with 500 μl serum-free media only (mock), or serum-free media with inoculum for to a final volume of 500 μl, and incubated at 37°C for 2 hours. After 2 hours, the supernatant was discarded and the cells were washed twice with PBS, prior to a 30min incubation at 37 °C with 500ul complete media with gentamicin (100ug/ml) to kill extracellular bacteria. Supernatant was discarded and cells were washed twice with PBS before lysis in 300ul of sterile de-ionised water. Complete lysis of samples was observed on the light microscope. Samples were then plated on BHI agar and incubated at 37°C overnight before colonies were counted, and CFU/ml calculated.

Oxford Nanopore Technologies (ONT) amplicon deep-sequencing

A library was prepared from 3 amplicons amplified with primer p1 and p5 and using ONT's Ligation sequencing kit (SQK-LSK109) with ONT's Native barcoding expansion kit (EXP-NBD104) according to the manufacturer's instructions (Protocol NBE_9065_v109_revH_23May2018). A final pooled library containing 676 ng was loaded onto a R9.4.1 flow cell. Sequencing was performed on a MinION device using all 1557 available pores for 72h. After completion of the sequencing run raw read files were base-called with ONT's Guppy-GPU v3.1.5 and subsequently demultiplexed using Guppy Barcoder v3.1.5. Adaptor and barcode sequences were finally trimmed from reads with Porechop v0.2.4. Between 1.0-1.5Gb were obtained per amplicon and the resulting read sets were deposited under BioProject PRJNA527676. The reads with forward and reverse primers and corresponding to the different *hsdMS* conformation have been identified with bbduk and seal program of the BBtools suite respectively (sourceforge.net/projects/bbmap/) and using the following commands: *bbduk.sh in=reads.fastq outm=reads_with_primer.fastq stats=primer_stats refstats=primer_refsats K=22 editdistance=2 qin=33*; *seal.sh in=reads_with_primer.fastq ref=hsdMS_references.fasta pattern=out_%.fa stats=hsdMS_stats refstats=hsdMS_refstats overwrite=t ambiguous=toss match=all qin=33.*

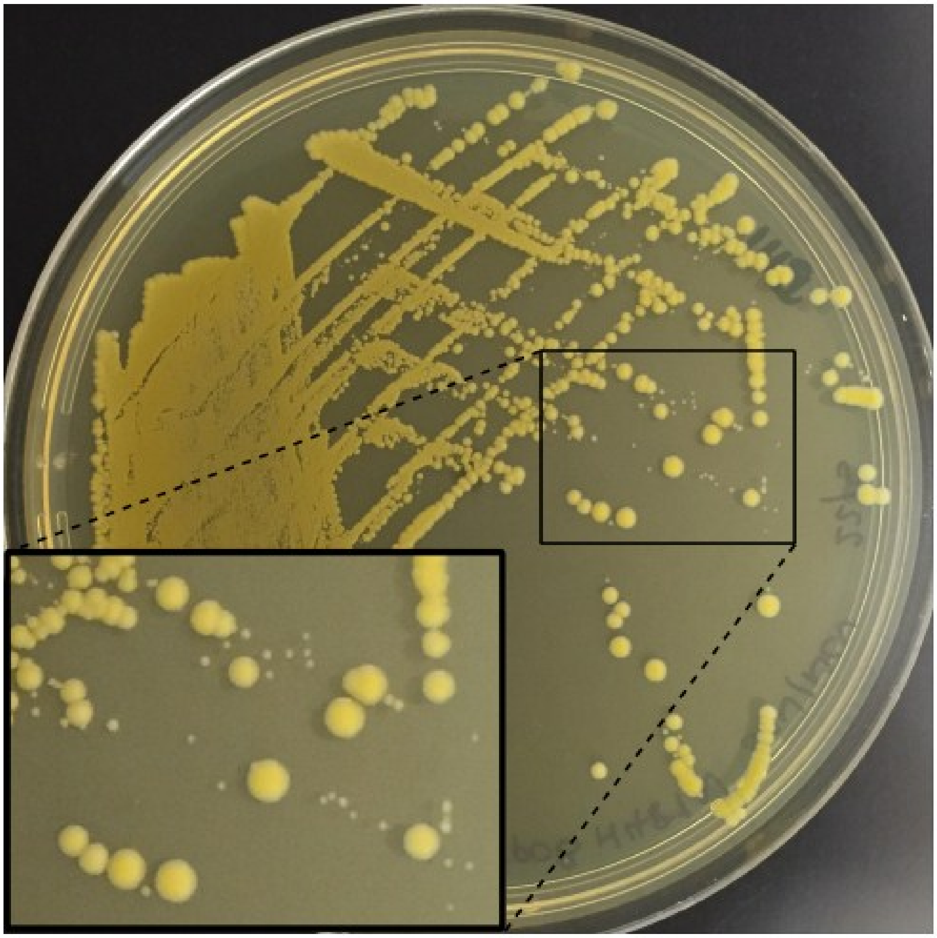

Fig. S1. Mixed colony morphology of the *rpoB*-H481N mutant of *S. aureus* NRS384. NRS384 *rpoB*-H481N was streaked on Brain Heart Infusion (BHI) agar plate and incubated 16 hours at 37°C.

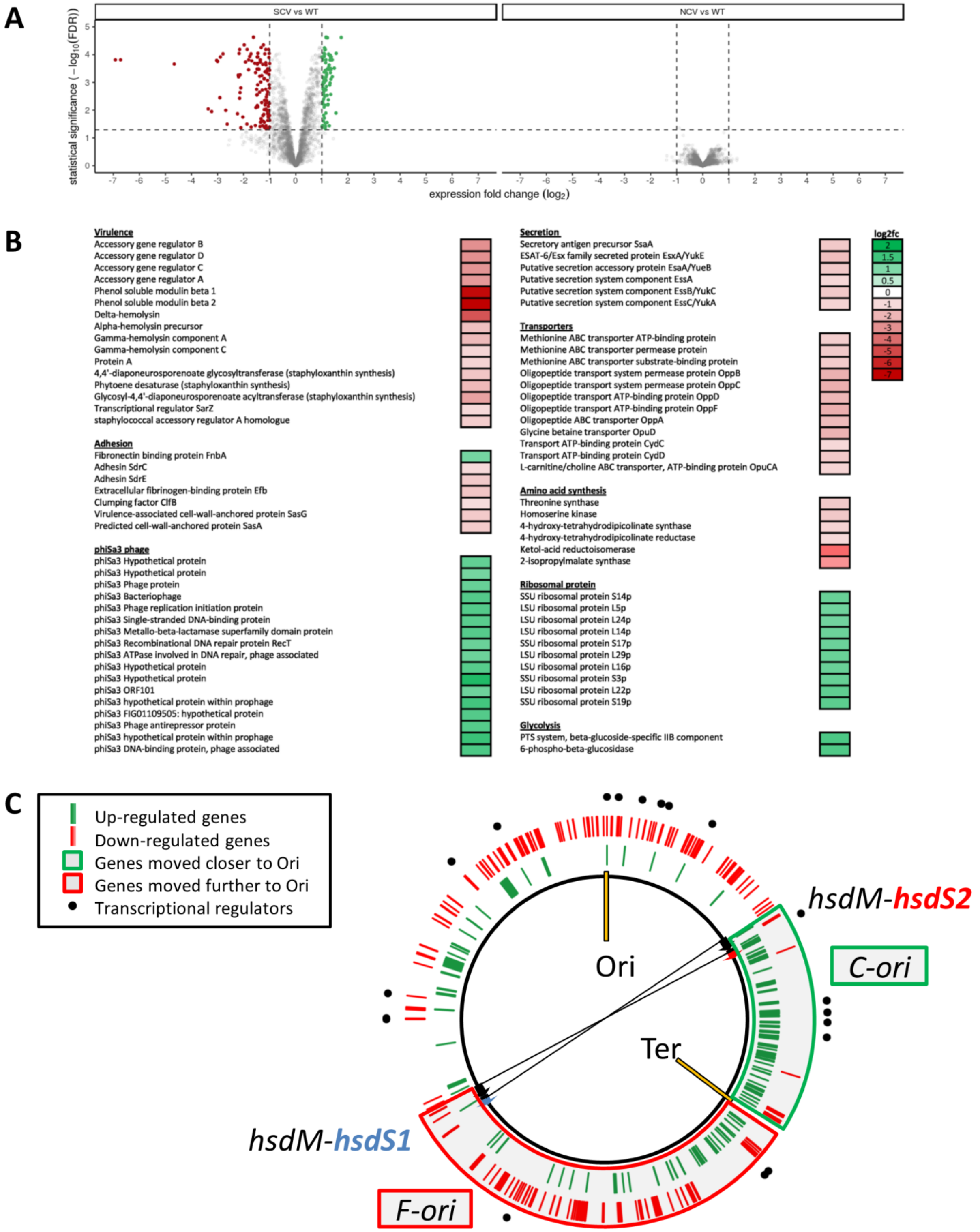

Locus	Up-regulated genes	Down-regulated genes	Up-regulation enrichment	Down-regulation enrichment	Significance (p value)
C-ori	100	8	**12.5**	0.1	**2.20E-16**
F-ori	58	123	0.5	**2.1**	**7.89E-05**
Whole chromosome	258	275	0.9	1.1	

Fig. S2 RNA-seq transcriptome comparisons of *S. aureus* WT, NCV and SCV. (A) Volcano plot representing significantly differentially expressed gene in the SCV (left panel) and NCV (right panel) compared to WT. Each dot represents a gene expression fold change (horizontal axis) with respect to statistical significance (vertical axis). Significantly up-regulated genes are colored green and down-regulated genes in red (FDR < 0.05 and |log2 fold change| > 1) (B) Selection of significantly differentially expressed gene in the SCV. The levels of transcriptional down-regulation or up-regulation are indicated by darker red or green boxes, respectively. Four biological replicates were assessed for each strain. (C) Replication-mediated gene dosage effect on transcription level. Up-regulated and down-regulated genes are indicated by green and red bars along the circular chromosome of the SCV (FDR < 0.05 and |fold change| > 1.5x). The genes moved closer to the replication origin by the chromosomal inversion (*C-ori* locus) present a significant enrichment of up-regulated genes whereas the genes moved further to the replication origin (*F-ori* locus) are significantly enriched with down-regulated genes.

Table S1. Detection and conservation of loci potentially promoting chromosomal inversion in 29 fully assembled S. aureus genomes.

**Detected inverted repeats loci**	**Number of strains**													**Number of strains with inverted repeats**	**Number of ST with inverted repeats**	**Comment**
	**ST8**	**ST5**	**ST239**	**ST1**	**ST22**	**ST105**	**ST133**	**ST151**	**ST250**	**ST254**	**ST36**	**ST398**	**ST93**			
rRNA	10	5	2	2	2	1	1	1	1	1	1	1	1	29/29	13/13	
hsdMS	10	5	2	2	0	1	1	1	1	1	1	0	1	26/29	11/13	***1***
IS1182	3	0	0	0	2	0	0	0	0	0	1	0	1	7/29	4/13	
IS1181	0	5	0	0	0	1	0	0	0	0	0	0	0	6/29	2/13	
ATPase phage phiSa3	0	0	2	0	0	1	0	0	0	0	0	0	0	3/29	2/13	
Transposase Tn554	0	1	2	0	0	0	0	0	0	0	0	0	0	3/29	2/13	
Transposase and ATPase	0	0	0	0	2	0	0	0	0	0	0	1	0	3/29	2/13	
IS256	1	0	2	0	0	0	0	0	0	0	0	0	0	3/29	2/13	***2***
Cell wall hydrolase	1	0	0	0	0	0	0	0	0	1	0	0	0	2/29	2/13	
Hypothetical protein	0	0	0	0	0	0	0	1	0	0	1	0	0	2/29	2/13	
SaPlm	0	1	0	0	0	0	0	1	0	0	0	0	0	2/29	2/13	***3***
Transposase	0	0	0	0	0	0	1	0	0	0	1	0	0	2/29	2/13	
ISSa1 IS30 family	0	0	0	0	0	0	0	0	0	0	1	1	0	2/29	2/13	
Phage tail protein	0	0	0	0	0	0	0	0	0	0	0	1	0	1/29	1/13	
Membrane protein	0	0	0	0	0	0	0	0	0	1	0	0	0	1/29	1/13	
DNA dommage inducible protein	0	0	0	0	0	0	1	0	0	0	0	0	0	1/29	1/13	
Phage structural protein	0	0	0	0	0	0	1	0	0	0	0	0	0	1/29	1/13	
Hypothetical protein	0	0	0	0	0	0	0	1	0	0	0	0	0	1/29	1/13	
Phage capsid protein	0	0	0	0	0	0	1	0	0	0	0	0	0	1/29	1/13	
Phage tail protein	0	0	0	0	0	0	1	0	0	0	0	0	0	1/29	1/13	
Group II intron	0	0	0	0	0	0	0	0	0	0	0	0	1	1/29	1/13	
Phage protein	0	0	0	0	0	0	0	0	0	0	0	1	0	1/29	1/13	
ISL3	0	0	0	0	0	0	0	1	0	0	0	0	0	1/29	1/13	
Phage antirepressor	0	0	0	0	0	0	1	0	0	0	0	0	0	1/29	1/13	
Hypothetical protein	0	0	0	0	0	0	0	0	0	1	0	0	0	1/29	1/13	
Pathogenicity island protein	0	0	0	0	0	0	0	1	0	0	0	0	0	1/29	1/13	

1 Promoting chromosomal inversion in this study

2 Promoting chromosomal inversion in OC8 strain (Wan et al. PLOS One, 2016)

3 Promoting chromosomal inversion in Mu50 strain (Cui et al. PNAS, 2012)

Table S2. Detection and conservation of ΦSa3 prophage in 7099 S. aureus genomes.

ST	Number of strains	Number of strains with *ΦSa3*	Percentage of strains with *ΦSa3*
All	7099	5711	80%
5	1791	1686	94%
8	1760	1656	94%
398	637	57	9%
105	544	512	94%
22	433	383	88%
30	174	143	82%
239	125	68	54%
45	119	105	88%
15	104	4	4%
36	84	81	96%
609	84	71	85%
1	60	57	95%
59	40	19	48%
25	33	29	88%
34	33	27	82%
250	33	30	91%
247	29	28	97%
228	28	20	71%
231	27	26	96%
72	26	20	77%
188	23	18	78%
12	21	16	76%
254	20	20	100%
Others	871	635	73%

Table S3. Primers used in this study.

**Primers**	**Sequence**	**Description**
ΦSa3_cf_F	CATTAGGGACTCCAAACCCAAT	qPCR of *ΦSa3* cirular form
ΦSa3_cf_R	AAAGTCCCTAAAAAGTCCCTAAAA	qPCR of *ΦSa3* cirular form
ΦSa3_ex_F	CAATAGTGCCAAAGCCGAAT	qPCR of *ΦSa3* excised form
ΦSa3_ex_R	CAGTTTTGTCCCACCCTGAT	qPCR of *ΦSa3* excised form
dnaA_F	GCAATTCAATGCCCATAACA	qPCR of *ΦSa3* reference gene
dnaA_R	GGCATGCATTAAATGGGTTT	qPCR of *ΦSa3* reference gene
p1	TCATGAATCCTATCCCAAT	qPCR of chromosomal inversion
p2	TTTTATTTTTCCTAATTACTCTATC	qPCR of chromosomal inversion
p3	TCTTTCCACTACCTATTTTGGT	qPCR of chromosomal inversion
p5	ACTAAAAGCTCTTCAGTCTCTTGTC	nanopore deep sequencing
IM119	CCTCACTAAAGGGAACAAAAGCTGGGTACCCCGTTCACAGTGATTGTGTATGG	construction of NRS384-Δ*ΦSa3*
IM120	CGACTCACTATAGGGCGAATTGGAGCTCGCCTGCTACATAGAATGTAGTAGG	construction of NRS384-Δ*ΦSa3*

Table S4. Accession numbers of fully assembled S. aureus genomes used for the detection of inverted repeats loci.

Strain accession	Strain name	ST
AJ938182.1	RF122	ST151
AM990992.1	ST398	ST398
AP009351.1	Newman	ST254
AP017377.1	OC8	ST8
BA000017.4	Mu50	ST5
BA000018.3	N315	ST5
LR130509.1	BPH2760	ST1
LR130511.1	BPH2819	ST5
LR130513.1	BPH2900	ST22
LR130515.1	BPH2947	ST239
LR130518.1	BPH2986	ST8
BX571856.1	MRSA252	ST36
BX571857.1	MSSA476	ST1
CP000046.1	COL	ST250
CP000253.1	NCTC 8325	ST8
CP000255.1	USA300 FPR3757	ST8
CP000736.1	JH1	ST105
CP001781.1	ED98	ST5
CP001996.1	ED133	ST133
CP002114.2	JKD6159	ST93
FN433596.1	TW20	ST239
HE681097.1	HO 5096 0412	ST22
NC_010079.1	USA300_TCH1516	ST8
NZ_CP007176.1	USA300-ISMMS1	ST8
NZ_CP007676.1	HUV05	ST8
NZ_CP007690.1	UA-S391	ST8
NZ_CP012119.1	USA300_2014.C01	ST8
https://figshare.com/collections/S_aureus_JKD6210_annotated_genome/2059784	JKD6210	ST5

SUPPLEMENTARY REFERENCES

1. Carter GP, et al. Genomic analysis of multiresistant staphylococcus capitis associated with neonatal sepsis. *Antimicrob Agents Chemother* **62**, e00898-18 (2018).

2. Proctor RA, et al. Staphylococcus aureus Small Colony Variants (SCVs): a road map for the metabolic pathways involved in persistent infections. *Front Cell Infect Microbiol* **4**, 99 (2014).

3. Law CW, Chen Y, Shi W, Smyth GK voom: Precision weights unlock linear model analysis tools for RNA-seq read counts. *Genome Biol* **15**, R29 (2014).

4. Monk IR, Tree JJ, Howden BP, Stinear TP, Foster TJ Complete Bypass of Restriction Systems for Major Staphylococcus aureus Lineages. *MBio* **6**, e00308-15 (2015).

5. Li H Aligning sequence reads, clone sequences and assembly contigs with BWA-MEM. *arXiv Prepr arXiv* (2013) doi:arXiv:1303.3997.

6. Carver T, Harris SR, Berriman M, Parkhill J, McQuillan JA Artemis: an integrated platform for visualization and analysis of high-throughput sequence-based experimental data. *Bioinformatics* **28**, 464–9 (2012).

7. Seemann T Prokka: rapid prokaryotic genome annotation. *Bioinformatics* **30**, 2068–2069 (2014).

8. Monk IR, Howden BP, Seemann T, Stinear TP Correspondence: Spontaneous secondary mutations confound analysis of the essential two-component system WalKR in Staphylococcus aureus. *Nat Commun* **8**, 14403 (2017).

9. Elek SD, Levy E Distribution of hæmolysins in pathogenic and non-pathogenic staphylococci.

J Pathol Bacteriol **62**, 541–554 (1950).

10. Liu GY, et al. Staphylococcus aureus golden pigment impairs neutrophil killing and promotes virulence through its antioxidant activity. *J Exp Med* **202**, 209–215 (2005).

11. Corriden R, et al. Tamoxifen augments the innate immune function of neutrophils through modulation of intracellular ceramide. *Nat Commun* **6**, 8369 (2015).

12. Bray NL, Pimentel H, Melsted P, Pachter L Near-optimal probabilistic RNA-seq quantification. *Nat Biotechnol* **34**, 525–527 (2016).
